# An Ovine Intestinal Organoid–Macrophage Co-Culture Model to Test the Effects of Ovine Colostrum Exosomes on Intestinal Barrier Function and Inflammation

**DOI:** 10.3390/ijms262311406

**Published:** 2025-11-25

**Authors:** Mahsa Dehnavi, Giulio Galli, Carlos García-Estrada, Rafael Balaña-Fouce, F. Javier Giráldez, Mercedes Alonso, Nuria Santos, Fernando Rozada, Sonia Andrés

**Affiliations:** 1 Instituto de Ganadería de Montaña (CSIC-Universidad de León), Finca Marzanas s/n, E-24346 Grulleros, León, Spain; mahsa.d@csic.es (M.D.); j.giraldez@eae.csic.es (F.J.G.); m.alonso@eae.csic.es (M.A.); n.santos@csic.es (N.S.); f.rozada@igm.ule-csic.es (F.R.); 2 Departamento de Ciencias Biomédicas, Facultad de Veterinaria, Universidad de León, Campus de Vegazana s/n, 24071 León, Spain; ggal@unileon.es (G.G.); c.gestrada@unileon.es (C.G.-E.); rbalf@unileon.es (R.B.-F.); 3 Instituto de Biomedicina (IBIOMED), Universidad de León, Campus de Vegazana s/n, 24007 León, Spain

**Keywords:** methionine, nutritional programming, three Rs, alternative methods, miRNA, epigenetics, organoids

## Abstract

Ovine colostrum exosomes obtained from nutritionally programmed dairy ewes (F0) may present modifications in microRNAs, thus having consequences for the intestinal barrier function and immunity parameters of lambs (F1). To test this hypothesis, colostrum exosomes from two ewe groups [F0-MET (nutritionally programmed ewes being fed methionine during early life) and F0-CTRL (ewes not supplemented with methionine during early life)] were sequenced to compare differences in the miRNAome. In addition, these exosomes were added to an in vitro co-culture in a Transwell chamber system consisting of ovine duodenum intestinal organoids and macrophages to assess the expression of genes encoding tight junction proteins in organoids and immunity parameters in macrophages. Finally, the concentrations of cytokines (e.g., IL-12 and IL-6) were assessed by ELISA kits in the supernatants of the chamber containing macrophages. According to the miRNAome, the expression of two miRNAs (e.g., oar_miR_376c_3p and oar_miR_432) was reduced in the colostrum exosomes obtained from dairy ewes nutritionally programmed with dietary supplementation of methionine during early life (F0-MET ewes). These changes did not seem to modify the expression of intestinal barrier and immune response marker genes when these exosomes were added to a co-culture of ovine intestinal organoids and macrophages. However, the levels of IL-12 produced by macrophages were reduced (*p* < 0.05), which suggests the inhibition of inflammatory pathways. Further studies using ovine colostrum exosomes obtained from nutritionally programmed ewes will help to clarify their potential to improve the health of suckling lambs.

## 1. Introduction

The “intestinal barrier” is a defensive system developed to block the passage of antigens, toxins, and microbial byproducts and simultaneously preserves the acquisition of tolerance against dietary antigens and the intestinal microbiota [1]. Disturbances in the mechanisms of barrier function favor the development of exaggerated immune responses and inflammatory conditions in the gastrointestinal tract, particularly in critical periods (e.g., preweaning phase) when the expression of genes encoding tight junction proteins (e.g., claudins, occludins, and desmosomal cadherins) may be compromised [2]. Previous studies in cattle have shown that functional microRNAs (miRNA) found in colostrum protect against lipopolysaccharide (LPS)-induced damage by downregulating inflammatory (*TNF-α*, *IL-6*, *IL-1β*) and apoptotic (*Bax*, *p53*, *Caspase-3*) genes and enhancing intestinal barrier genes (*TJP1*, *CLDN1*, *MUC2*) [3]. However, methionine supplied during early postnatal life in ewe lambs (suckling period of F0) has shown potential to modify DNA methylation [4], thus changing gene expression throughout the whole life. These changes might have consequences for the miRNAs contained in colostrum exosomes during the lactation period (F0 adulthood) [5], with implications for the intestinal barrier function and health of the offspring (F1), as demonstrated in humans and pigs [6,7]. To our knowledge, no studies have tested this hypothesis in an ovine model.

To test this hypothesis, and to explain the mechanisms involved in the observed effects, the miRNAome of colostrum exosomes produced by dairy sheep being fed methionine during early life (F0) was sequenced, before adding these vesicles to an in vitro co-culture model comprising ovine intestinal organoids (functionally characterized in Galli et al. [8]) and macrophages. The purpose of this study was to assess the effects of methionine supplementation during the suckling period in ewe lambs on miRNA expression in colostrum exosomes produced during the lactation period (F0 adulthood), as well as their impacts on the intestinal barrier function of the offspring (F1). Preliminary results point towards potential anti-inflammatory properties of colostrum exosomes produced by nutritionally programmed dairy ewes.

## 2. Results

### 2.1. Animal Performance of F0 Ewes and Milk Fatty Acid Profile

Methionine included in the milk replacer (DM basis) during the early postnatal life (suckling period) of ewe lambs did not improve feed efficiency or milk production during lactation (F0). However, permanent changes mediated by DNA methylation and epigenetic marks were observed in lipid metabolism, with increased percentages of *n*3, *n*6, and long-chain fatty acids in the milk produced by these animals. All results are presented and discussed in detail in Dehnavi et al. [4].

### 2.2. miRNAome of Colostrum Exosomes of F0 Ewes

**Figure 1:**
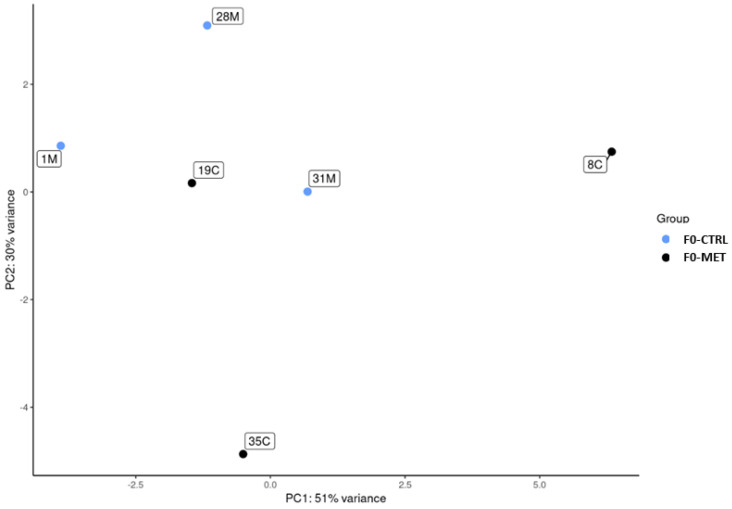
The principal component analysis (PCA) of miRNA expression in ewe colostrum exosomes is shown via blue dots for the F0-MET group and black dots for the F0-CTRL group. PC1 and PC2 account for 51% and 30% of the total variance, respectively.

The sequencing quality control of miRNAs contained in colostrum exosomes (F0) revealed appropriate fragment sizes, and FastQC (e.g., read counts, GC content, and Q20/Q30 scores) and confirmed the adequacy of these parameters. After pre-processing, the fragment size was mainly between 20 and 25 nucleotides, and the percentage of quality-filtered reads of miRNA (e.g., after discarding rRNA, tRNA, artefacts, and unknowns) was 51.5%. Expression profiles were compared by principal component analysis (Figure 1), which resulted in two different clusters (both sets of samples separated) according to PC1 (51% variance explained) and PC2 (30% variance explained).

Finally, differential expression analysis with DESeq2 identified two downregulated miRNAs (Table 1) in the colostrum exosomes of F0-MET dams compared to the control ones (F0-CTRL).

**Figure 2:**
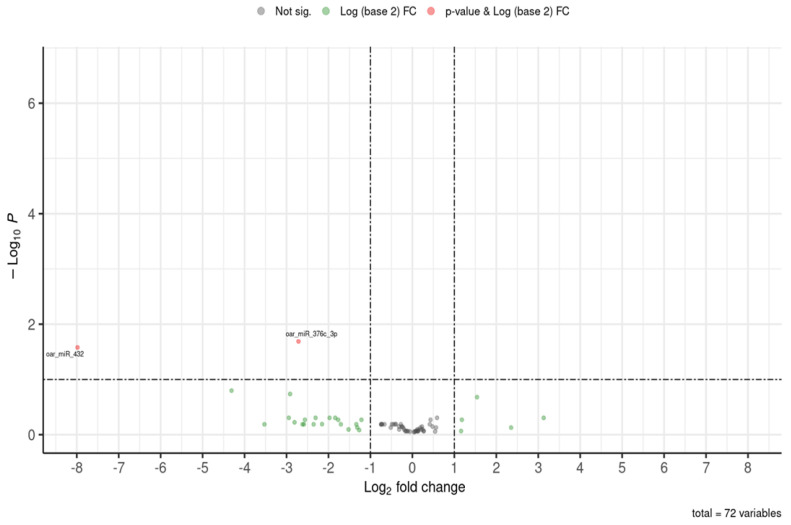
Volcano plot showing comparison of expressed miRNAs in colostrum exosomes of methionine-supplemented ewes (F0-MET) and controls (F0-CTRL) during early postnatal life. The plot represents statistical significance (*p*-value, Y-axis) versus the magnitude of change (log2FC, X-axis). Grey dots represent neither statistical significance nor differences in expression. Green dots represent no statistical significance but changes in expression. Red dots represent both statistical significance and differences in expression. Downregulated miRNAs are shown on the left, and upregulated miRNAs are shown on the right side.

**Figure 3:**
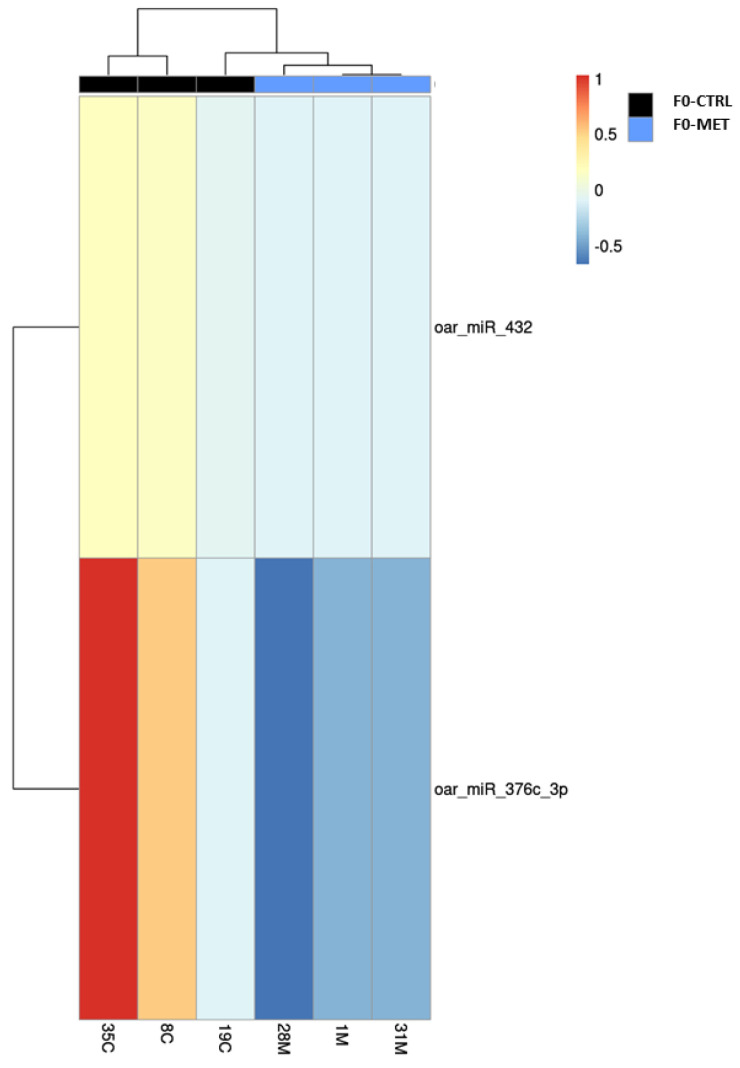
Heatmap showing the most repressed genes in the colostrum exosomes of the two conditions (F0-CTRL and F0-MET). Two significantly downregulated miRNAs were detected in the colostrum exosomes of F0-MET dairy sheep (31M and 1M). Colors indicate the level of expression from downregulation (blue) to upregulation (red). Dendrograms indicate similarity between samples. The filters applied were log2FC < −1 or >1 and q-value < 0.1.

These results are visualized in a volcano plot (Figure 2) and a heatmap (Figure 3), showing distinct expression patterns based on significant fold changes and q-values. Other annotated miRNAs whose differential expression between both groups did not reach the significance level (FDR, q-value, or adjusted *p*-value ≤ 0.1) can be found in Supplementary Table S1.

### 2.3. Effects of Exosomes on Co-Cultures of Ovine Intestinal Organoids and Macrophages

**Figure 4:**
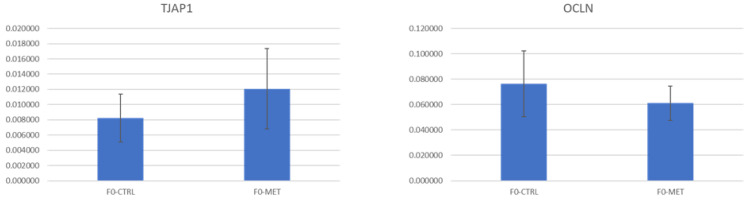
Expression of genes (efficiency-corrected target quantity, N0) encoding tight junction proteins (e.g., *TJAP1* and *OCLN*) in ovine intestinal organoids after 4 h of incubation with colostrum exosomes obtained from methionine-supplemented ewes (F0-MET) or controls (F0-CTRL) during early postnatal life.

**Figure 5:**
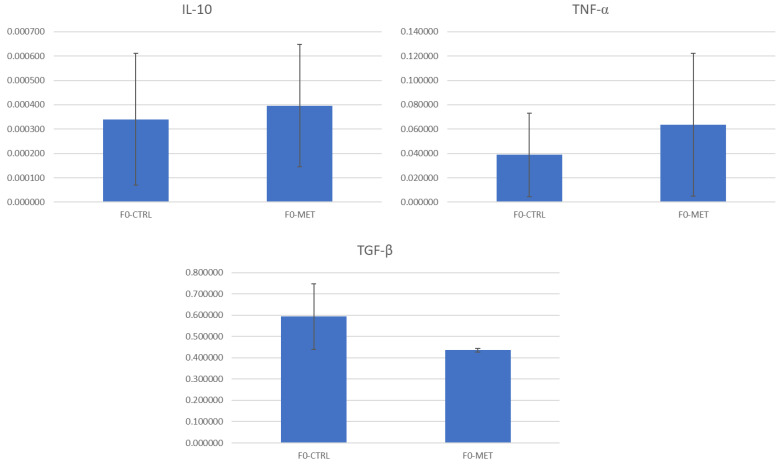
Expression of genes (efficiency-corrected target quantity, N0) encoding anti-inflammatory (e.g., *IL-10* and *TGF*-β) and pro-inflammatory cytokines (e.g., *TNF*-α) in ovine macrophages after 4 h of incubation with colostrum exosomes obtained from methionine-supplemented ewes (F0-MET) or controls (F0-CTRL) during early postnatal life.

The expression of genes encoding tight junction proteins (e.g., *TJAP1* and *OCLN*) in ovine intestinal organoids and immune response markers (e.g., *TNF*-α, *IL*-10, and *TGF*-β) in macrophages after 4 h of incubation with colostrum exosomes was assessed by RT-qPCR. Based on the standard errors shown in the bar plots, there were no statistically significant differences in the expression of any of these genes promoted by the colostrum exosomes of F0-MET ewes when compared to the control (F0-CTRL) group (Figure 4 and Figure 5).

The results of ELISA measurements in the supernatant obtained from macrophage chamber revealed significantly lower levels of IL-12 (a pro-inflammatory cytokine) in the co-cultures incubated with colostrum exosomes obtained from F0-MET ewes when compared to the F0-CTRL group (Table 2, *p* = 0.048). However, no effects on IL-6 levels between groups were detected.

## 3. Discussion

This study examined the long-term effects caused by methionine supplementation in ewe lambs during early postnatal life on the miRNAome of the colostrum exosomes obtained during the lactation period (adulthood). The results showed that methionine supplementation during early life promoted permanent changes that caused the downregulation of two genes encoding two different miRNAs found in colostrum exosomes during the lactation period: oar_miR_376c_3p and oar_miR_432. The first miRNA whose expression was significantly downregulated (e.g., oar_miR_376c_3p) has been related to the posttranscriptional regulation of genes involved in fertility, fecundity, and prolificacy traits in sheep [9,10]. Moreover, a human melanoma model demonstrated that the downregulation of mir-376a and mir-376c may contribute to *IGF1R* (e.g., insulin-like growth factor 1 receptor) overexpression [11], which has been associated with insulin resistance [12]. The second miRNA downregulated in the colostrum exosomes of F0-MET dairy sheep (e.g., oar_miR_432) has been implicated in both fat differentiation and the expression of bone morphogenetic protein 2 (e.g., *BMP2*) in ovine preadipocytes [13]. Overall, the F0-MET colostrum exosomes exhibited lower expression levels of miRNAs associated with insulin resistance and bone formation, thus corroborating some phenotypical traits already described for these F0-MET ewes [4] and therefore the potential of these molecules to be used as biomarkers of nutritional programming events [5].

Moreover, the potential effects of these exosomes on the intestinal barrier function and inflammatory responses of the offspring were evaluated in vitro using a co-culture of ovine intestinal organoids and macrophages. Previous authors have demonstrated the potential of both bovine milk exosomes and extracellular vesicles (EVs) to increase the expression of genes involved in the intestinal epithelial barrier, even under LPS-induced damage conditions [3,14]. However, according to the results observed in the present study, the colostrum exosomes of ewes being fed methionine during early life (F0-MET) had no significant effects on the expression levels of intestinal barrier-related genes (e.g., *TJAP1* and *OCLN*, *p* > 0.05) when compared to the control group F0-CTRL. This indicates that changes promoted by methionine supplementation during the early life of ewe lambs in the miRNAome of the colostrum exosomes produced during the lactation period do not directly improve epithelial barrier function at a gene transcription level when compared to the control group (F0-CTRL).

In contrast, the secretion of the inflammatory cytokine IL-12 was significantly reduced (*p* < 0.05) when co-cultures were treated with F0-MET colostrum exosomes, while IL-6 was unaffected. IL-12 plays an important role in the activation of the Th1 response, thus increasing systemic inflammatory responses [14,15]. Therefore, its decrease may suggest innate immune modulation by F0-MET exosomes when compared to the control group, although it is not clear whether this is a positive or negative effect. In fact, this point should be clarified under the conditions of an experimental challenge (e.g., *E.coli* or LPS), an objective that was beyond the scope of this study. In any case, these results are also consistent with existing evidence that exosomes from bovine milk can control inflammatory responses by blocking the TLR4–NF-κB pathway [15]. Additionally, in a mouse cell model of LPS-induced intestinal damage, EVs derived from bovine colostrum significantly reduced the expression of inflammatory genes such as *TNF-α*, *IL-6*, and *IL-1β* [3], whereas others (e.g., *TNF-α*, *IL-10*, and *TGF-β* genes) were not affected [14,15]. The discrepancies between studies might be explained by either the different models used (e.g., inflammatory or non-inflammatory conditions, types of cells, etc.), differences in exosome biosynthesis, or different animal species, i.e., ovine and cattle.

Overall, the results obtained validate the initial hypothesis that methionine supplementation during the suckling period in ewe lambs modifies miRNAs in colostrum exosomes produced during the lactation period (F0 adulthood); these changes show a potential anti-inflammatory impact on the intestinal barrier function of the offspring. Nevertheless, the limitations of the present study must be also considered, with the lack of a specific ovine culture medium for organoid differentiation being the most relevant. Although ovine intestinal organoids were finally produced using the IntestiCult™ OGM Mouse Medium (Ref. 06000; STEMCELL Technologies, Saint-Egrève, France), the number of mature organoids after passing was low (approximately 100 organoids per well), thus limiting the amount of RNA extracted per well and also the number of genes whose expression was measured by RT-qPCR. Nowadays, the industry is making progress to optimize the medium in order to support both the expansion and differentiation of intestinal organoids in a single culture system. Therefore, further studies using ovine colostrum exosomes are guaranteed to clarify their effects on cell co-cultures and therefore their potential to improve the health of suckling lambs; this knowledge may contribute to improving the health and viability of the offspring (F1), thereby decreasing the incidence of some diseases in newborn animals (e.g., diarrhea) and the use of antibiotics, which would be aligned with the challenges (i.e., sustainable food production) recognized by the European Common Agricultural Policy and the European Green Deal.

## 4. Materials and Methods

### 4.1. Colostrum Sample Collection, Exosome Isolation, and miRNAome Sequencing

All details of the Assaf dairy ewes [F0, experimental flock of the Instituto de Ganadería de Montaña (CSIC, León, Spain)] and the proximate composition of the milk obtained are given in full in Dehnavi et al. [4]. Briefly, a control group of newborn Assaf lamb ewes (F0-CTRL, n = 17) was fed ad libitum with a commercial milk replacer (Cordevit Calostrado, Leches Maternizadas S.A., León, Spain). In contrast, the other group (F0-MET, n = 17) received the same milk replacer supplemented with 0.1% D,L-methionine (Rhodimet^®^ NP 99, Addiseo, Commentry, France) on a DM basis [16]. After weaning (approximately 45 days of age), all F0 ewe lambs were housed together and reared under identical conditions, being offered ad libitum a complete pelleted diet (CPD) formulated according to their nutritional requirements [17]. Once they were 9 months old, all F0 ewe lambs were synchronized by the administration of progestogens via intravaginal sponges containing 20 mg of flugestone acetate (Chronogest, MSD Animal Health, Salamanca, Spain). After 14 days, the sponges were removed and 500 international units of gonadotropin (Foligon 6000, MSD Animal Health, Salamanca, Spain) was immediately administered intramuscularly. Finally, the ewes were artificially inseminated. Colostrum samples were collected on the first day of lactation, frozen in 2 mL Eppendorf tubes, and stored at −80 °C.

Then, exosomes were isolated from the colostrum using a commercial kit [Total Exosome Isolation Reagent (from other body fluids), Invitrogen^TM^, Gangseo-gu, Republic of Korea] according to the manufacturer’s instructions. Small RNAs, including miRNAs, were isolated from colostrum exosomes obtained from 6 animals (F0-MET, n = 3 and F0-CTRL, n = 3) with a miRNeasy serum/plasma kit (Qiagen Cat No./ID: 217184, Venlo, The Netherlands), according to the manufacturer’s protocol. Libraries of small RNAs were constructed using the NEXTFlex Small RNA-Seq Kit v4 (Illumina-compatible) (PerkinElmer, Waltham, MA, USA), according to the manufacturer’s guidelines. Size selection was performed by targeting fragments that were 150–180 bp in size using BluePippin (Sage Science, Beverly, MA, USA). Size-selected libraries were then PCR-amplified with indexed sequencing primers and finally sequenced on a NovaSeq X Plus System (Illumina, San Diego, CA, USA) for 150 × 2 cycles in high-output mode, with target coverage of 20 M reads for each library. The statistical and bioinformatics pipeline used to determine the differentially expressed miRNAs was implemented according to the workflow described below.

In brief, quality control (QC) and metric calculation for pre-trim raw FastQ and processed BAM files were performed with the FastQC software, version 0.11.9. The alignment of the reads to the reference genome assembly (Ovis aries-ARS-UI_Ramb_v3.0 (https://www.ncbi.nlm.nih.gov/datasets/genome/GCF_016772045.2/, accessed on 5 September 2024) was performed using the miRDeep2 software (version 2.0) with sequences smaller than 18 bp being discarded. Different miRNAs were identified using miRBase v. 22, and raw read counts were quantified and organized in a table, including all analyzed samples. Differential expression analysis using the DESeq2 software (version 1.46.0) was implemented with the above-mentioned count table using the Benjamini–Hochberg procedure to control the false discovery rate (FDR, q-value, or adjusted *p*-value ≤ 0.1). A negative log2 fold change indicated lower expression of a miRNA in the treatment group (F0-MET) when compared to the control (F0-CTRL) group.

### 4.2. Isolation of Ovine Intestinal Crypts and Intestinal Organoid Culture

The use of in vitro organoids is aligned with the three R principles to replace, reduce, and refine the use of animals under experimental conditions. Therefore, the generation of duodenum intestinal organoids was carried out as previously described [8]. Briefly, the duodenum of a healthy 4-month-old male lamb was harvested at the slaughterhouse; then, tissue was cut longitudinally, cut into 2 mm pieces, and rinsed with PBS until the supernatant was clear, and tissue pieces were incubated for 15 min at room temperature in 25 mL of Gentle Cell Dissociation Reagent (Ref. 100-0485; STEMCELL Technologies) on a rocking platform at 20 rpm. After sedimentation, the supernatant was discarded. Tissue fragments were washed with 10 mL cold PBS (2–8 °C) + 0.1% BSA, and dissociated cells were filtered through four 70 µm cell strainers (Ref. 431751; Corning^®^, New York, NY, USA). After centrifugation (300× *g*, 5 min, 2–8 °C), pellets were resuspended in 10 mL PBS + 0.1% BSA and then centrifuged again (200× *g*, 3 min, 2–8 °C) and resuspended in 10 mL cold DMEM/F-12, HEPES (Ref. 11330032; Gibco, Thermo Fisher Scientific, Grand Island, NY, USA). Intestinal crypt pellets were resuspended in 150 µL room-temperature IntestiCult™ OGM Mouse Medium (Ref. 06000; STEMCELL Technologies) with 100 U-mg/mL penicillin–streptomycin solution 100X (Ref. SV30010, Cytiva) and 50 mg/mL gentamycin (Ref. 15750037; Gibco, Thermo Fisher Scientific). Fifty-microliter droplets were plated in 24-well plates (Ref. 142475; Thermo Fisher Scientific) with Geltrex™ LDEV-Free Reduced Growth Factor Basement Membrane Matrix (Ref. A1413202; Gibco, Thermo Fisher Scientific). After 10 min of polymerization at 37 °C in a 5% CO_2_ incubator, 500 µL of room-temperature medium was added to each well. Organoids were cultured for 7–10 days, with the medium refreshed every 2–3 days, and monitored by phase-contrast microscopy for lumen formation and spherical morphology.

**Figure 6:**
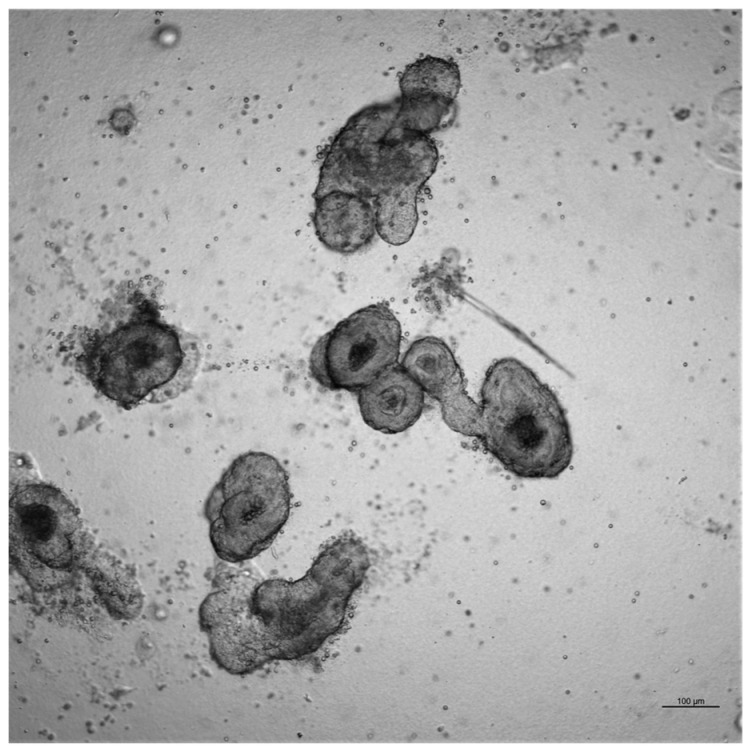
Ovine intestinal organoid (3rd passage, day 5). Scale bar = 100 μm.

For passaging, the medium was removed, and organoids were dissociated with 800 µL Gentle Cell Dissociation Reagent (Ref. 100-0485; STEMCELL Technologies, Saint-Egrève, France) per well, mechanically disrupted, and incubated at room temperature on a rocking platform at 20 rpm for 10 min. The suspension was centrifuged (300× *g*, 5 min, 2–8 °C), and the pellet was resuspended in 10 mL ice-cold DMEM/F-12, HEPES (Ref. 11330032; Gibco, Thermo Fisher Scientific), followed by centrifugation (200× *g*, 5 min, 2–8 °C). Organoids were split 1:2 to 1:8 and seeded in IntestiCult™ OGM Mouse Medium with antibiotics (Ref. 06000; STEMCELL Technologies), mixed with Geltrex™ (Ref. A1413202; Gibco, Thermo Fisher Scientific), and replated in 24-well plates for continued culture (Figure 6).

### 4.3. Monocyte Isolation and Macrophage Differentiation

Moreover, 100 mL of blood was collected by jugular venipuncture from a healthy 6-month-old male lamb using 10 mL vacutainer tubes with heparin. Briefly, monocytes were isolated by magnetic cell separation (MACS) using LS separation columns (Ref. 130-042-401; Miltenyi Biotec^®^, Madrid, Spain) and CD14 MicroBeads (Ref. 130-050-201; Miltenyi Biotec^®^, Madrid, Spain), according to the protocol described by Arteche-Villasol et al. [18]. Then, the pellet was diluted 1:2 in trypan blue for monocyte counting using a standard hemocytometer Neubauer chamber, and the cells were adjusted to 10^6^ cells/mL in 10 mL of macrophage colony-stimulating factor medium. Finally, the cells were transferred to 75 cm^2^ culture flasks (Ref. 156499; Thermo Fisher Scientific) and incubated at 37 °C in a humidified atmosphere with 5% CO_2_ for 10 days to allow the differentiation of monocytes into macrophages.

### 4.4. Exosome Addition to Co-Culture of Ovine Intestinal Organoids and Macrophages

**Figure 7:**
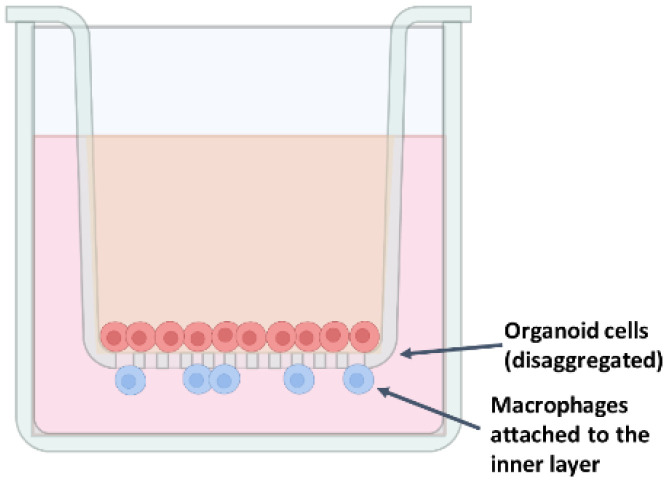
A well in the Corning Transwell system used to co-culture ovine intestinal organoids and macrophages.

A 12-well Transwell chamber system with 0.4 µm polyethylene terephthalate (membranes—Ref. 3470; Corning^®^) was used to co-culture duodenum intestinal organoids and macrophages in order to test the effects of colostrum exosomes obtained from ewes being fed methionine during early postnatal life on the intestinal immunity response, integrity barrier, and inflammation. Inserts were first pre-coated with 75 µL Geltrex™ (Ref. A1413202; Gibco, Thermo Fisher Scientific) in 1.425 µL ice-cold PBS and incubated at 37 °C in a humidified atmosphere with 5% CO_2_ for 1 h, and then the coating solution was aspirated from the surface of the insert for organoid seeding. Macrophages were trypsinized with pre-warmed trypsin–EDTA (0.05%) (Ref. 25200072; Gibco, Thermo Fisher Scientific), and incubated at 37 °C, 5% CO_2_ for 4 min. The cells were centrifugated (900× *g*, 7 min, 4 °C) in 9 mL of pre-warmed PBS, and the pellet was resuspended in 5 mL M-CSF medium. Macrophages were counted, and 2 × 10^4^ cells/well were added to 6 mL monolayer medium containing IntestiCult™ Human OGM Medium (Ref. 06010; STEMCELL Technologies), 50 µg/mL gentamicin, 1% penicillin–streptomycin, and 10 µM Y-27632 (Ref. 72302; STEMCELL Technologies). A total of 500 µL of the suspension was seeded into the basolateral chamber of the plate. Organoids from the given passage were dissociated with 7 mL pre-warmed trypsin–EDTA (0.05%) (Ref. 25200072; Gibco, Thermo Fisher Scientific) for 5 to 7 min, neutralized with ice-cold DMEM/F-12, HEPES (Ref. 11330032; Gibco, Thermo Fisher Scientific), centrifuged (200× *g*, 5 min, 2–8 °C), and resuspended in 1200 µL monolayer medium containing IntestiCult™ Human OGM Medium (Ref. 06010; STEMCELL Technologies), 50 µg/mL gentamicin, 1% penicillin–streptomycin, and 10 µM Y-27632 (Ref. 72302; STEMCELL Technologies), and 100 µL of the suspension was seeded into the apical chamber (Figure 7). Monolayers were maintained for 5–10 days, with medium changes every 2–3 days, until confluence was achieved.

Then, colostrum exosomes of 6 different F0 ewes (3 F0-CRTL and 3 F0-MET ewes; already sequenced for miRNA) were added to the Transwell (2 wells per ewe). A total of 50 µg of exosome protein, quantified using a Pierce^TM^ BCA Protein Assay kit (Thermo Fisher Scientific, Inc.) according to the manufacturer’s instructions and diluted in 100 µL of monolayer medium (see above), was added to the apical chamber of each well. After 4 h of incubation, cell samples and supernatants from both chambers (e.g., intestinal organoids and macrophages) were collected separately for RNA extraction and ELISA protocols to assess the cellular responses of both intestinal organoids and macrophages, as described below.

### 4.5. RNA Extraction and Real-Time Quantitative Polymerase Chain Reaction (RT-qPCR)

Briefly, total RNA was extracted from intestinal organoids and macrophages using the GeneMATRIX Universal RNA Purification Kit (EURx Ltd., Gdansk, Poland). The RNA quantity was measured using the QuantiFluor^®^ RNA System and a Quantus^TM^ Fluorometer (Promega) and the RNA integrity number determined using Bioanalyzer 2100 (Agilent Technologies, Santa Clara, CA, USA). The total RNA was reversed-transcribed to cDNA using the Invitrogen™ SuperScript™ VILO™ Master Mix, according to the manufacturer’s instructions. The RNA was used as a template for real-time reverse transcription PCR analysis. Finally, the expression of genes encoding inflammation in macrophages (e.g., cytokines such as *TNF-α*, *IL-10*, and *TGF-β*) and intestinal barrier integrity function proteins in intestinal organoids (e.g., *TJAP1* and *OCLN*) was assessed by RT-qPCR using the pairs of primers described in Table 3.

The Ct values were normalized according to the expression of the *B2M* gene (housekeeping gene) and the results expressed as the efficiency-corrected target quantity (N0) calculated by the LinRegPCR program.

### 4.6. Cytokine Measurement in Macrophage Culture Supernatants

To detect and quantify the levels of ovine IL-12 p70 and ovine IL-6, the macrophage cell culture supernatant was collected, and cytokine production was measured using the Invitrogen^TM^ Ovine IL-12 p70 and Invitrogen^TM^ Ovine IL-6 ELISA kits, according to the manufacturer’s instructions. Briefly, the absorbance was measured at 450 nm after adding the stopping solution. Based on the standard curve and the four-parameter algorithm, concentration values were reported in pg/mL.

### 4.7. Statistical Analysis

Data are presented as the mean ± SE, and the significance of differences was determined by Student’s *t* test. *p* values less than 0.05 were considered significant.

## 5. Conclusions

Under the conditions of the present study, it can be concluded that 0.1% D,L-methionine included in the milk replacer (DM basis) during the early postnatal life of ewe lambs (F0) causes permanent changes responsible for differences in the miRNAome of the colostrum exosomes obtained during the lactation period. These changes may show a significant effect in reducing intestinal inflammation but not improving the epithelial barrier structure. Further in vitro and in vivo experiments will be required to clarify whether these changes in the miRNA exosomes of colostrum F0 dams being fed methionine during early life show potential to improve the gut health of the offspring (F1).

## Figures and Tables

**Table 1:** miRNA differential expression analyses of colostrum exosomes obtained from dairy ewes nutritionally programmed with 0.1% methionine during the suckling period (F0-MET).

miR Name (miRBASE)	log2FC *	*p*-Value	q-Value
oar_miR_376c_3p	−2.715	<0.05	0.021
oar_miR_432	−7.983	<0.05	0.026

* a negative log2FC means lower expression of the miRNA in the treatment (F0-MET) when compared to the control (F0-CTRL) group.

**Table 2:** Levels of ovine IL-12 p70 and IL-6 measured by ELISA in the supernatants of co-cultures of ovine intestinal organoids and macrophages incubated with colostrum exosomes obtained from methionine-supplemented ewes (F0-MET) or controls (F0-CTRL) during early postnatal life.

Cytokine	F0-CTRL	F0-MET	SED	*p*-Value
IL-6	0.071	0.072	0.003	0.961
IL-12	9.39	2.95	2.008	0.048

**Table 3:** Primers used for RT-qPCR amplification and the study gene of expression in ovine intestinal organoids and macrophages.

Gene	NCI Reference	Forward Primer	Reverse Primer	Product Size
*OCLN*	XM_015101257.4	ATGCCTTTTGGAGTCATGCC	CACAACACTGGCAAACATGC	139 bp
*TJAP1*	XM_027958379.3	TGCTCAAGTGCAACAAGTCC	TGTTTCCGAACCATGTCCTG	82 bp
*TNF-α*	NM_001024860.1	CCAGAGGGAAGAGCAGTCC	GGAGCGCTGATGTTGGCTAC	126 bp
*IL-10*	NM_001009327	CCAGGATGGTGACTCGACTAG	TGGCTCTGCTCTCCCAGAAC	75 bp
*TGF-β*	NM_01009400	GAAGTCTAGCTCGCACAGCA	CACGTGCTGCTCCACTTTTA	133 bp
*B2M*	XM_060418694.1	TTCATTGTGCCTGCCTTTCC	TGCAAAACACCCTGACCAAG	138 bp

## Data Availability

The miRNA data used in this study are available via the following DIGITAL.CSIC accession number: 390474 (http://hdl.handle.net/10261/390474, accessed on day 18 March 2025).

## Supplementary Materials

The following supporting information can be downloaded at: https://www.mdpi.com/article/10.3390/ijms262311406/s1.

## Abbreviations

The following abbreviations are used in this manuscript.

BSA	Bovine serum albumin
CLDN1	Claudin-1
CTRL	Control
ELISA	Enzyme-linked immunosorbent assay
IL-1β	Interleukin 1β
IL-6	Interleukin 6
IL-10	Interleukin 10
IL-12	Interleukin 12
LPS	Lipopolysaccharide
M-CSF	Macrophage colony-stimulating factor
MET	Methionine
miRNAs	MicroRNAs
MUC2	Mucin 2
PBS	Phosphate-buffered saline
NF-κB	Nuclear factor-kappa B
OCLN	Occludin
PBMCs	Peripheral blood mononuclear cells
RT-qPCR	Real-time quantitative polymerase chain reaction
TGF-β	Transforming growth factor beta
TJAP1	Tight junction protein 1
TLR4	Tool-like receptor 4
TNF-α	Tumor necrosis factor
